# Abdominal Symptoms in General Practice, a Case Control Study

**Published:** 1987-05

**Authors:** Paul R. Bond

**Affiliations:** General Practitioner, The Surgery, Coronation Road, Downend, Bristol

## Abstract

A survey was carried out of three hundred general practice patients who were questioned about recurrent abdominal pain or discomfort and other related symptoms. Patients who responded positively to the questionnaire fell into three main groups.

Twenty two patients (7%) were found to have previously diagnosed irritable bowel syndrome. A further twenty patients (7%) had similar symptoms but had not sought medical help for them. Finally there were twenty eight (9%) patients with abdominal pain due to miscellaneous organic diseases.

Age-sex matched controls were selected from the two hundred and thirty patients who responded negatively to assess the rate of consultation and the rate of prescribing benzodiazepines and antidepressants.


### Abdominal Symptoms in General Practice A Case Control Study

**Dr Paul R. Bond MRCP, MRCGP**
General Practitioner, The Surgery, Coronation Road, Downend, Bristol

### ABSTRACT

A survey was carried out of three hundred general practice patients who were questioned about recurrent abdominal pain or discomfort and other related symptoms. Patients who responded positively to the questionnaire fell into three main groups.

Twenty two patients (7%) were found to have previously diagnosed irritable bowel syndrome. A further twenty patients (7%) had similar symptoms but had not sought medical help for them. Finally there were twenty eight (9%) patients with abdominal pain due to miscellaneous organic diseases.

Age-sex matched controls were selected from the two hundred and thirty patients who responded negatively to assess the rate of consultation and the rate of prescribing benzodiazepines and antidepressants.

### INTRODUCTION

It has previously been shown that abdominal symptoms are common in the general population. Estimates of the incidences in people not seeking health care range from 20.6% (1) to 17.1% (2) and in the latter study half of those with symptoms had not sought medical advice. It has also been shown that certain symptoms are present more commonly in irritable bowel patients than in patients with organic diagnoses, suggesting that a definitive diagnosis of the irritable bowel syndrome can be made rather than relying on exclusion criteria (3).

The purpose of this study was to find the incidence of various abdominal symptoms in a general practice population who had sought health care for other reasons.

An attempt was made to quantify the workload these patients present to their G.P. by looking at consultation rates. I also made an attempt to quantify psychiatric morbidity by noting how many patients were, or had been taking benzodiazepine tranquilizers or antidepressants.

### METHOD

Three hundred consecutive patients attending one general practitioner were asked to complete a questionnaire whilst waiting to be seen. They brought the questionnaire into the consultation and certain points were clarified if necessary. The patients were mainly caucasian from mixed suburban and rural background. The questionnaire contained an initial stem question enquiring as to whether the patient had had recurrent abdominal pain or discomfort in the past year. Patients who answered 'Yes' to this were then asked about the nature of the pain in five further questions to find out:

1) Was the pain situated below the navel.
2) The frequency of the pain.
3) Was the pain affected by bowel actions.
4) Were motions looser with the onset of pain.
5) Were motions more frequent with the onset of pain.

All patients were asked about whether they had:

1) Abdominal pain after food.
2) Bloating or distention.
3) Presence of mucus in the motions.
4) Frequency of laxative use.
5) A feeling of incomplete evacuation.
6) Had any of these symptoms been mentioned to their G.P. or had they seen a specialist.

Finally the notes were reviewed to assess the number of consultations in the past year and to see if the patient was taking or had ever taken antidepressants or benzodiazepine tranquilizers. Statistical significance of the results from each group was assessed using the Chi-squared test.

### RESULTS

Seventy patients (23%) admitted to recurrent abdominal pain in the previous year. These patients fell into three main categories. Twenty two (7%) had previously diagnosed irritable bowel syndrome, twelve diagnosed by the G.P. without referral and the remaining ten confirmed by a specialist. Twenty-eight patients (9%) had miscellaneous organic causes for their pain and these are summarised in Table I. A further twenty (7%) had recurrent abdominal pain but had never consulted their G.P. about it.

I could find no statically significant differences among the three groups as regard to the nature of the pain. (The results are summarised in Table II).

From the remaining two hundred and thirty patients without recurrent pain age-sex matched controls were randomly selected to assess the relative frequency of the other abdominal symptoms. (The results are summarised in Table III).

Consultation rates show no significant change from the controls although there was a tendency for the patients with irritable bowel and organic disease to consult more frequently.

**Table 1**

**Organic Diagnoses in 28 Patients**

| | |
|---|---|
| Gallstones | 6 |
| Gynaecological | 4 |
| Duodenal ulcer | 3 |
| Haemorrhoids or fissure | 3 |
| Hiatus hernia | 3 |
| Diverticulitis | 3 |
| Constipation | 1 |
| Rectal prolapse | 1 |
| Chronic pancreatitis | 1 |
| Crohns disease | 1 |
| Cause unknown despite investigation | 2 |
| | 28 |

**Table 2**

**The Nature of the Pain in each Subgroup**

| *Nature of pain* | *Irritable bowel* *No. 22* | *Non-complainers* *No. 20* | *Organic* *No. 28* |
|---|---|---|---|
| Below navel | 20 (90.1) | 16 (80) | 22 (78.6) |
| Frequency | | | |
| At least once a day | 3 (13.6) | 0 | 5 (17.9) |
| Once a day—once a week | 3 (13.6) | 8 (40) | 9 (35.7) |
| Less than once a week | 16 (72.8) | 12 (60) | 13 (46.4) |
| Pain affected by bowel action | 9 (40.9) | 10 (50) | 8 (28.6) |
| Bowels looser with onset of pain | 15 (69.6) | 15 (75) | 19 (71.4) |
| Bowels more frequent with onset of pain | 15 (69.2) | 13 (65) | 15 (53.6) |

Percentages in brackets.
No group differed significantly from another.

**Table 3**

**Associated symptoms in each group compared with controls**

| *Associated feature* | | *Irritable bowel/ control* | *Non-complainers/ control* | *Organic/ control* |
|---|---|---|---|---|
| Pain after food | | 13/2 * | 14/7 ‡ | 18/8 † |
| Bloating | | 18/4 * | 17/8 † | 22/7 * |
| Mucus in motions | | 6/1 ‡ | 3/1 N.S. | 10/3 ‡ |
| Laxative use | | 3/3 N.S. | 6/4 N.S. | 8/2 ‡ |
| Incomplete evacuation | | 12/5 ‡ | 15/5 † | 19/3 * |
| Benzodiazepine | Current | 0/0 | 0/0 | 1/3 N.S. |
| | Ever | 7/4 N.S. | 7/6 N.S. | 9/4 N.S. |
| Antidepressant | Current | 1/1 N.S. | 1/1 N.S. | 3/0 N.S. |
| | Ever | 3/2 N.S. | 4/0 ‡ | 5/4 N.S. |
| Consultation rates | | 8.1/5.4 | 6.1/6.7 | 8.8/7.1 |

*=P 0.001 †=P 0.01 ‡=P 0.05

Benzodiazepine and tranquilizer prescribing showed no difference from the controls save for the non-complainers who appear to have been given more anti-depressants in the past. However this may be a spurious result.

### DISCUSSION

Although this sample of the population is selected it appears to show the same incidence of abdominal symptoms as the other studies in a population not seeking medical advice.

With the figures presented here the average G.P. with a list size of 2,200 patients has about 150 patients with confirmed irritable bowel syndrome and a similar number of patients with similar symptoms who have, for one reason or another, not mentioned it to him. He will see one of each group in an average morning surgery. Almost a quarter of his patients will have had recurrent abdominal symptoms in the previous year.

This questionnaire, as designed, would not detect the group of irritable bowel patients with painless diarrhoea. Esler and Goulston (4) found this group of patients had higher anxiety and neuroticism scores than either the general population of the subgroup with predominantly abdominal pain. This may explain why there was no excess of psychotropic prescribing for the irritable bowel cohort.

What makes a person with intermittent abdominal pain present his symptoms to a doctor, thereby becoming a patient is a mystery (5). The group of non-complainers probably comprise a majority of patients with irritable bowel symptoms. Some may have dyspepsia (6) and some may be harbouring organic conditions.

Despite the fact that Hislop (7) found evidence of affective disorder in the majority of his 67 patients and 80% of the 56 patients he treated with antidepressants improved significantly, the patients with irritable bowel syndrome in this study were not more likely to have been treated with psychotropic drugs than the control group. Of the ten patients with irritable bowel syndrome who had seen a specialist only one had been given an antidepressant and only three had been given benzodiazepines.

It appears that the hard core of "introspective psychological cripples" (8) which attend gastroenterology outpatients are just the tip of a large iceberg of patients with gastrointestinal symptoms, and many of these consider their symptoms too trivial to report to their doctor.

### ACKNOWLEDGEMENTS

I would like to thank Dr Richard Harvey for his advice and support.

(continued on page 50)

### Abdominal symptoms in General Practice *(Continued from page 34)*

### REFERENCES

1. THOMPSON, WE and HEATON, KW (1980) Functional bowel disorders in apparently healthy people. *Gastroenterology* **79**, 283–288.
2. DROSSMAN, DA, SANDLER RS, McKEE, DC and LOUITZ, AJ (1982) Bowel patterns among subjects not seeking health care. *Gastroenterology* **83**, 529–534.
3. MANNING, AP, THOMPSON, WG, HEATON, KW and MORRIS AF (1978) Towards positive diagnosis of the irritable bowel. *B.M.J.* **2**, 653–654.
4. ESLER, MD and GOULSTON, KJ (1973) Levels of anxiety in colonic disorders. *New England Journal of Medicine* **288**, 16–20.
5. ALPERS, DH (1981) Irritable bowel—still more questions than answers. *Gastroenterology* **80**, 1068–1069.
6. GEAR, MWL and BARNES, RJ (1980) Endoscopic studies of dyspepsia in a general practice. *B.M.J.* **I**, 1136–7.
7. HISLOP, IG (1971) Psychological significance of the irritable colon syndrome. *Gut* **12**, 452–457.
8. EDITORIAL *Lancet* (1984) Dec 1st: **2**, 8414:1249–50.

